# Increased Attentive Use Is Linked to More Idiosyncratic Functional Connections

**DOI:** 10.1523/JNEUROSCI.2178-25.2026

**Published:** 2026-08-06

**Authors:** Pinar Demirayak, Leland L. Fleming, Pauline Stewart, Rachel Chua, Kristina M. Visscher

**Affiliations:** ^1^Civitan International Research Center, University of Alabama at Birmingham, Birmingham, Alabama 35294; ^2^Department of Neurobiology, Heersink School of Medicine, University of Alabama at Birmingham, Birmingham, Alabama 35233; ^3^Department of Psychiatry, McLean Hospital/Harvard Medical School, Boston, Massachusetts 02478

**Keywords:** fMRI, functional connectivity, lesion projection zone, macular degeneration, plasticity, preferred retinal locus, resting state

## Abstract

Experience is thought to modify neural connections to adapt the network to be more optimal for the environment. Given the brain's complexity, multiple network changes could each move the system toward optimality. Standard approaches examine each connection independently; these studies have often shown considerable interindividual variability and modest effects (Marek et al., 2022). Here, we take a different strategy, determining how a whole-brain connection pattern differs from the typical pattern, that is, how “idiosyncratic” the pattern is. We examined how the idiosyncrasy of whole-brain connection patterns varies with frequency of the use of that part of cortex for attention-demanding tasks, focusing on central versus peripheral vision in healthy individuals (who use central vision more frequently for attention-demanding tasks). We found that the whole-brain pattern of functional connections to the cortical representations of central vision is idiosyncratic, whereas patterns of connections to representations of peripheral vision were very similar person to person in healthy vision controls (14 females, 9 males). In a second set of analyses, we examined the brains of people with central vision loss (11 females, 10 males) who use a portion of peripheral vision [the preferred retinal locus (PRL)] more frequently for attention-demanding tasks in their daily lives. The cortical representation of the PRL exhibits more idiosyncratic connections, compared with a control brain region or compared with the same brain region in matched healthy vision controls. These results are consistent with the hypothesis that increased attentive use of a brain area results in idiosyncratic patterns of whole-brain connections.

## Significance Statement

We found that increased attentive use of a brain region results in more idiosyncratic patterns of connections of that region to the rest of the brain. Our findings support the view that the primary visual cortex retains the capacity for plasticity well beyond the critical period and that these adaptations are idiosyncratic to the individual's experiences. This approach suggests that tailoring personalized rehabilitation plans for individuals with retinal diseases may be more effective than a “one-size-fits-all” approach. More generally, it offers a promising framework for investigating brain plasticity in both typical and clinical populations, especially in the context of sensory loss and compensatory adaptation.

## Introduction

Comedian Jamie MacDonald, who is blind, said “Some people, they see disability as *one* thing. Blindness has infinite combinations of psychological and physical impacts on people… Blind people, we're all different. We're like snowflakes. There are not two of us the same. And if a lot of us fall, people panic” (MacDonald, 2025). MacDonald's humorous quote brings home a point that is often ignored when studying brain plasticity: the brain's response to changes in experience is idiosyncratic. As researchers, we typically average responses among individuals, forgetting that each participant's experience is like a snowflake.

Interindividual variability in brain connectivity reflects the interplay between common neural architectures and unique individual factors. While the large-scale organization of brain networks is broadly shared across people (Damoiseaux et al., 2006; Power et al., 2011), extensive prior work shows that functional connections also carry idiosyncratic signatures (Mueller et al., 2013; Gratton et al., 2018). These idiosyncratic features may arise from personal experience or from other factors, and these idiosyncrasies can relate to individual behavioral traits (Finn et al., 2015; Gratton et al., 2018). Because experience is inherently idiosyncratic, the connectivity changes that accompany it are unlikely to be uniform across individuals. This idiosyncrasy is not evenly distributed across the brain: regions involved in sensory processing, such as the early visual cortex, generally exhibit lower interindividual variability than higher-order association areas (Mueller et al., 2013; Wang et al., 2015). However, the extent to which variability of connection patterns emerges following experience remains poorly understood. To address this, we tested whether brain regions representing frequently attended portions of the visual field—central vision in individuals with healthy sight—show more idiosyncratic versus more stereotyped patterns of whole-brain functional connectivity (FC).

People with healthy vision preferentially attend to the fovea due to its high spatial resolution (Silver and Kastner, 2009; Carrasco, 2011). Vision subserved by the fovea and macula of the retina is critical for reading, recognizing faces, and scrutinizing whatever objects or scenes a person attends. Occipital and frontoparietal attention networks are more active when stimuli are presented foveally (as opposed to peripherally), reflecting a natural bias of attentional systems toward central vision (Schneider et al., 2004). Thus, brain regions associated with central vision habitually undergo more attentive use than those associated with peripheral vision.

Central vision loss due to macular degeneration (MD) damages the macula while sparing much of the peripheral retina (Wong et al., 2014) with severe consequences for everyday life (Mitchell and Bradley, 2006). Individuals with MD often adopt a preferred retinal locus (PRL) in the intact peripheral retina as a new fixation point for their daily attended activities (Fletcher and Schuchard, 1997; Cheung and Legge, 2005). Thus, habitual attention shifts from the fovea to the PRL. Due to the retinotopic organization of the early visual areas in humans, central vision loss is an ideal model to study experience-dependent changes in the cortex.

Resting-state FC reveals both shared and individual-specific features of brain organization, reflecting the balance between canonical large-scale networks and idiosyncratic connection patterns. Connections within networks, such as the default mode network or the dorsal attention network, show strikingly consistent strong connections in almost all individuals (Gratton et al., 2018). Yet alongside this stereotypy, there are unique patterns of fine-grained functional connections (Finn et al., 2015; Gratton et al., 2018). Fine-grained patterns of connectivity vary substantially between people, capturing the influence of individual anatomy, development, and experience (Mueller et al., 2013; Braga and Buckner, 2017), resulting in patterns of connections that are unique to an individual and are sometimes called “connection fingerprints.” Building on this framework, we asked whether the functional connections of visual field representations—particularly those habitually engaged for attention—change in the context of central vision loss. Our data are consistent with the hypothesis that brain regions that experience “more attentive use” show more idiosyncratic patterns of whole-brain connections.

## Materials and Methods

### Participants

MRI data were acquired from 44 [21 MD (11 females and 10 males), 23 control (14 females and 9 males)] participants as a part of the Connectomes in Human Diseases Macular Degeneration Plasticity project (Visscher and Stewart, 2024). MD participants had been previously diagnosed by an ophthalmologist as having bilateral MD. Our inclusion criteria for the MD group include having a diagnosis for at least 2 years prior to being enrolled in the study, having bilateral dense scotomas at the center of the fovea, and not having any psychiatric or neurological diseases. All MD participants underwent visual acuity testing, using the Early Treatment Diabetic Retinopathy Study (ETDRS) test (Ferris et al., 1982), macular integrity assessment (MAIA; CenterVue), and optical coherence tomography to confirm that all MD participants have foveal scotomas in both eyes and low visual acuity. Demographic information of MD participants is summarized in Table 1. Written informed consent was obtained from all participants prior to enrollment in the study. Data were collected in accordance with the ethical standards under the oversight of the University of Alabama at Birmingham (UAB) Institutional Review Board.

**Table 1.** Demographic information of participants with MD

Participant ID	NDA Participant ID	Sex	Age (y)	Age onset(y)	Time since onset (y)	Diagnosis	Education (y)	Better eye	Visual acuity (ETDRS)	BCEA (deg) 63	PRL Ecc (degree)
MDP004	NDARMD204VRJ	F	73	56	17	AMD	18	OD	20/32	7.2	4.85
MDP005	NDAR_INVR8JH6WGH	M	22	15	7	JMD	14	OS	20/125	9.7	10.85
MDP006	NDAR_INVWUEYLKYY	F	50	46	4	AMD	16	OS	20/160	6.8	4.3
MDP008	NDARET638JNX	F	70	50	20	AMD	15	OS	20/160	54.5	12.9
MDP014	NDARYN951XM7	M	68	34	34	JMD	18	OS	20/400	10.1	10.13
MDP016	NDAR_INVAX6B2Y33	M	55	42	13	AMD	15	OD	20/32	9.3	17.1
MDP020	NDARPF637HEG	F	79	72	7	AMD	11	OS	20/80	4	2.45
MDP021	NDAR_INVR7U690Y7	M	27	14	13	JMD	13.5	OS	20/200	36.3	9.16
MDP022	NDAR_INV284UKW5H	F	83	70	13	AMD	14	OD	20/100	18.8	6.98
MDP023	NDAR_INVV64G9T4X	F	35	19	16	JMD	18	OD	20/400	12	8.19
MDP044	NDAR_INVJR547GW1	M	91	81	10	AMD	12	OS	20/50	3.9	2.99
MDP047	NDARTN260UH7	M	77	71	6	AMD	16	OD	20/32	11.4	5.14
MDP050	NDARYD612JZE	M	83	73	10	AMD	20	OS	20/125	12.2	7.79
MDP065	NDARTA691AGE	F	61	38	23	AMD	12	OS	20/160	27.4	12.56
MDP100	NDAR_INVZZ4X0EKD	M	80	75	5	AMD	16	OS	20/40	8.1	2.12
MDP117	NDAR_INV586K3JYG	M	88	82	6	AMD	16	OS	20/32	9.7	5.19
MDP122	NDAR_INVJKLY20FZ	F	70	19	51	JMD	16	OD	20/400	16.9	12.37
MDP123	NDAR_INVR3386E5L	F	70	66	4	AMD	18	OS	20/200	5.5	6.15
MDP126	NDAR_INVVYA60NKF	M	83	65	18	AMD	14	OD	20/80	2.7	3.33
MDP132	NDAR_INVXYNGV921	F	93	59	34	AMD	17	OS	20/63	11.5	3.63
MDP174	NDARJG572NXA	F	57	47	10	AMD	14	OS	20/400	84.4	9.46

ETDRS, Early Treatment Diabetic Retinopathy Study; AMD, age-related macular degeneration; JMD, juvenile macular degeneration, BCEA, bivariate contour ellipse area.

### Definition of preferred and unpreferred retinal locus on retinal images

**Figure 1.**
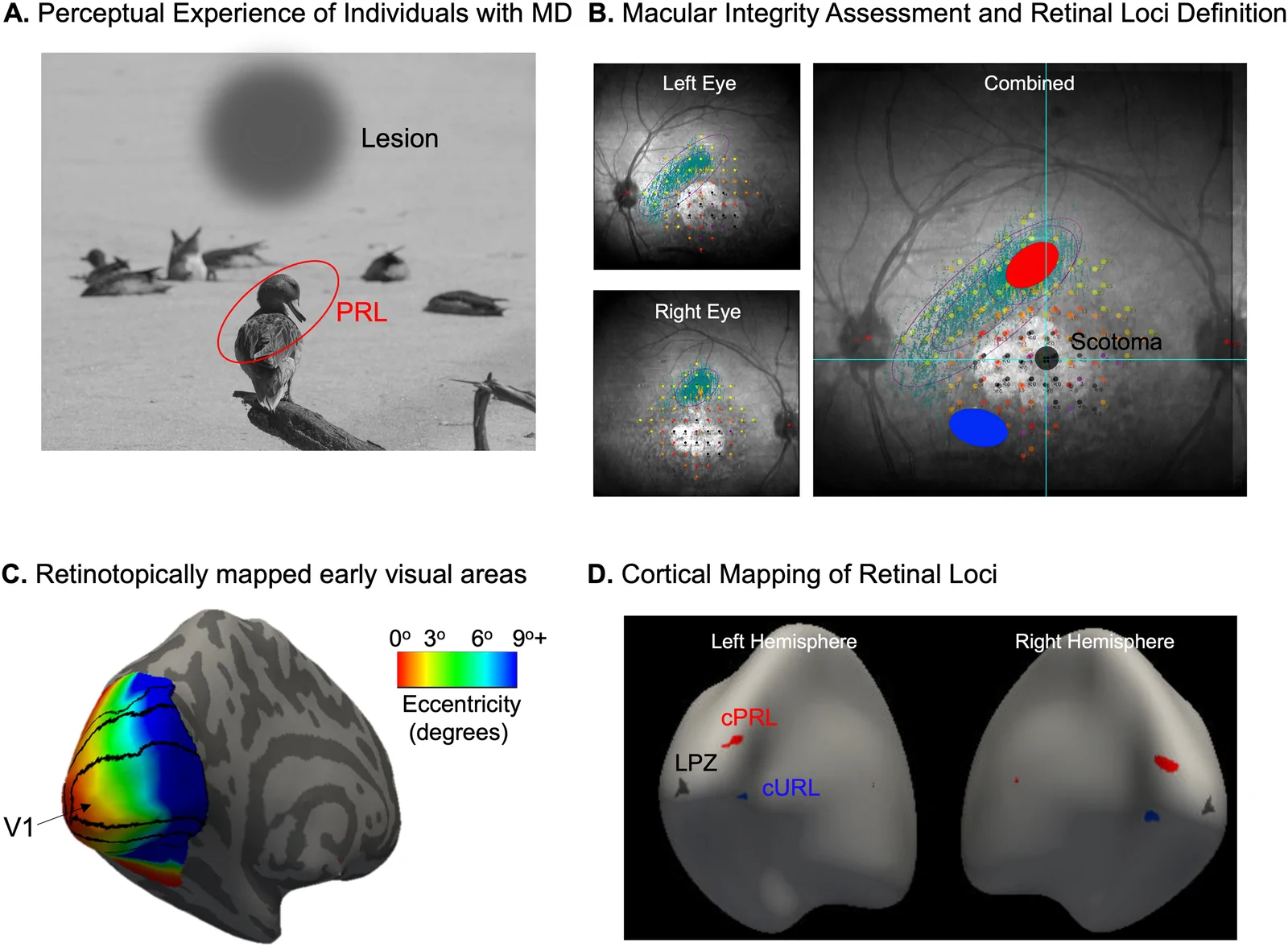
Identifying retinal loci (lesion, PRL, and URL) and their cortical projections. ***A***, Representation of the perceptual experience of individuals with MD. ***B***, Fundus photograph of the retina from one eye of a representative participant with central vision loss. The grayscale background shows the anatomy of the retina. Microperimetry results are shown in the grid of colored dots. The colors of dots represent the threshold for detection of a spot of light at that location on the retina. Black dots indicate no light detection. Teal dots represent retinal locations to which the participant orients a fixation target. Cyan ellipse encloses 63% of participants' fixations. Note that the right eye has a tighter fixation window than the left. The location of these fixations on the better eye is defined as the PRL. The URL is defined in an equally eccentric location to the PRL, with similar visual detection and retinal health. ***C***, Illustration of the anatomic atlases of eccentricity, polar angle, and sigma mapping in early visual areas. Eccentricity is represented across the cortex with central vision at the occipital pole. ***D***, Based on the anatomic atlases, we define the cortical projections of the scotoma (LPZ, shown in dark gray), cPRL (shown in red), and cURL(shown in blue) in V1. The LPZ ROI was defined as the 100 vertices with the lowest assigned eccentricity values in both hemispheres in V1.

We estimated the retinal location at which the MD patients tended to primarily fixate (the PRL) from a MAIA, outputs of which are illustrated in Figure 1. The PRL (Fig. 1*A*) was defined based on the cloud of fixation locations over time (Fig. 1*B*). The center of the PRL was defined as the center of the cloud, and the boundary was defined as the bivariate contour ellipse area (BCEA) that included 63% of fixation locations (Steinman, 1965). PRL size (BCEA that encompasses 63% of fixation locations) was calculated automatically by the MAIA device. PRL eccentricity for each MD participant was measured manually in Photoshop as pixel distance from the fovea to the center of the BCEA multiplied by the pixel-per-degree conversion factor supplied by the MAIA manufacturer. We also defined a control retinal region that we refer to here as the “unpreferred retinal locus” or URL*.* This region was defined by identifying a region on the retina outside of the lesion with the same eccentricity as the PRL region. MAIA images and regions of interest (ROIs) on the retina and on the cortex are shown in Supplementary Figures (Figs. S1–S11).

### MRI data acquisition

All images were acquired on a 3 T Siemens Prisma scanner using a 64-channel head-coil at the UAB using protocols based on those of the Human Connectome Project (Glasser et al., 2016; Visscher and Stewart, 2024). High-resolution 3D anatomical scans were obtained (T1-weighted (T1w); repetition time (TR), 2,400 ms; echo time (TE), 2.22 ms; field of view [FOV(ap,rl,fh)], 208 × 208 × 144 mm; voxel size, 0.8 mm isotropic; flip angle (FA), 8°). T1w images were reacquired if the initial acquisition was of poor quality. Resting-state functional scans (eyes open) were acquired in total darkness (T2*-weighted, TR, 800 ms; TE, 37 ms; FA, 52°; voxel size, 2 mm isotropic; echo spacing, 0.58 ms; multiband acceleration factor, 8), resulting in 420 volumes per scan. Because the retinal input to patients with MD and controls is different, we examine data here that was collected during rest in total darkness, which equates the retinal contrast in both groups. To ensure complete darkness, the investigators blocked out all possible sources of visible light in the room (windows, waveguides, lights on equipment, using black felt) prior to the start of the resting-state scan. Participants were instructed to relax and keep their eyes open until the scan was complete. For one participant (MDP044, who had MD), lights-on resting-state data were used because that participant did not complete the study and we did not acquire resting-state data during darkness in that participant. An infrared eye-tracking system camera (M0203 Livetrack AV, Cambridge Research Systems), which does not emit visible light, was used to monitor participants during scanning to ensure that their eyes remained open during the scanning session. The resting-state data were acquired in four runs and each run took 5 min 36 s.

### MRI data preprocessing

Raw data fMRI data files were formatted according to the Brain Imaging Data Structure standard (Gorgolewski et al., 2016) to enable use with open-source preprocessing pipelines. Initial first-pass quality control was performed through manual inspection of the data using MRIQC (Esteban et al., 2017). Preprocessing of the fMRI data was performed using fMRIPrep 1.2.5 (Esteban et al., 2019), which is based on Nipype 1.1.6 (Gorgolewski et al., 2011).

#### Anatomical data preprocessing

T1w images were corrected for intensity nonuniformity and skull-stripped. Brain surface reconstruction was performed using recon-all from FreeSurfer v6.0.0 (Dale et al., 1999). Spatial normalization to the ICBM 152 Nonlinear Asymmetrical template version 2009c was performed through nonlinear registration with antsRegistration (ANTs 2.2.0; Avants et al., 2008), using brain-extracted versions of both T1w volume and template. Brain tissue segmentation of cerebrospinal fluid (CSF), white matter (WM), and gray matter (GM) was performed on the brain-extracted T1w using FSL 5.0.9 (Zhang et al., 2001).

#### Functional data preprocessing

Functional MRI data preprocessing in fMRIPrep was carried out as follows: First, a reference volume and its skull-stripped version were generated using fMRIPrep. The BOLD reference was then coregistered to the T1w reference using bbregister (FreeSurfer), which implements boundary-based registration (Greve and Fischl, 2009). Coregistration was configured with 9 df to account for distortions remaining in the BOLD reference. Head-motion parameters concerning the BOLD reference (transformation matrices and six corresponding rotation and translation parameters) are estimated before any spatiotemporal filtering (Jenkinson et al., 2002). BOLD runs were slice-time corrected using 3dTshift from AFNI 20160207 (Cox and Hyde, 1997). The slice-time corrected BOLD time series were resampled onto their original, native space by applying a single composite transform to correct for head motion and susceptibility distortions. These resampled BOLD time series will be referred to as preprocessed BOLD. The BOLD time series were also resampled to MNI152NLin2009cAsym standard space. First, a reference volume and its skull-stripped version were generated using fMRIPrep. Several confounding time series were calculated based on the preprocessed BOLD: framewise displacement (FD), DVARS, and three region-wise global signals. FD and DVARS were calculated for each functional run, using their implementations in *Nipype* 1.1.6 (Gorgolewski et al., 2011) following the definitions by Power et al. (2014). The three global signals are extracted from the CSF, the WM, and the whole-brain masks.

Additionally, a set of physiological regressors was extracted to allow for component-based noise correction (Behzadi et al., 2007). Principal components are estimated after high-pass filtering the preprocessed BOLD time series (using a discrete cosine filter with 128 s cutoff) for the two CompCor variants: temporal (tCompCor) and anatomical (aCompCor). Six tCompCor components are then calculated from the top 5% variable voxels within a mask covering the subcortical regions. This subcortical mask is obtained by heavily eroding the brain mask, which ensures it does not include cortical GM regions. For aCompCor, six components are calculated within the intersection of the aforementioned mask, and the union of CSF and WM masks is calculated in T1w space after their projection to the native space of each functional run (using the inverse BOLD-to-T1w transformation). The head-motion estimates calculated in the correction step were also placed within the corresponding confounds file. All resamplings were performed with a single interpolation step by composing all the pertinent transformations (i.e., head-motion transform matrices, susceptibility distortion correction when available, and coregistrations to anatomical and template spaces). Gridded (volumetric) resamplings were performed using antsApplyTransforms, configured with Lanczos interpolation to minimize the smoothing effects of other kernels (Lanczos, 1964). Nongridded (surface) resamplings were performed using mri_vol2surf (FreeSurfer).

#### Motion correction

Motion artifacts during fMRI scanning are known to produce substantial effects on FC data (Power et al., 2012; Van Dijk et al., 2012). To mitigate such confounds, we performed motion scrubbing on the preprocessed data from fMRIPrep using the XCP engine workflow (Ciric et al., 2018) using a FD threshold of 0.5 mm. Following the preprocessing procedure, the data were converted into a template space (fsLR_32k cifti-space) using a combination of Ciftify (Dickie et al., 2019) and the Connectome Workbench (Marcus et al., 2011).

### ROI definitions on the cortex

ROIs in the primary visual cortex (V1) were defined based on maps of the visual field that were acquired from retinal imaging, coupled with standard atlases of visual regions (Benson and Winawer, 2018), as described in our previous work (Defenderfer et al., 2021; Fig. 1). PRL and URL areas on the retina were defined as described in above, Definition of preferred and unpreferred retinal locus on retinal images. We used a cortical mapping method to define participant-specific cortical projections of the PRL and URL (referred to as the cPRL and cURL, respectively). These were created for each MD participant based on their retinal image and for each control participant based on every MD participant's retinal image. Details of the cortical mapping method were described in Defenderfer et al. (2021) and Defenderfer et al. (2021). In brief, we created binary LPZ, cPRL, and cURL ROIs in retinal space using Photoshop. Standard retinotopic atlases including eccentricity, polar angle, and sigma were transferred to each participant's cortical surfaces through the Python library neuropythy (Benson and Winawer, 2018). These atlases assign visual eccentricity, polar angle, and sigma values to each vertex in early visual areas. Each pixel in the MAIA image for the PRL and URL was assigned an eccentricity and polar angle value based on pixel distance from the center of the fovea multiplied by a pixel to visual degree conversion factor obtained from the MAIA microperimeter. For each vertex in V1, the retinotopic coordinates were extracted from the retinotopic atlas, and the pixel from the MAIA image with the closest eccentricity and polar angle values was assigned to that vertex. If that pixel was located in any given retinal ROI (PRL, URL), that vertex was added to that cortical ROI label (cURL, cURL). All ROIs were mapped on individuals' native cifti space and then converted into 32k standard space.

Because our MD participants have bilateral central vision scotomas of extremely different sizes, to keep the size of the ROI corresponding to central vision consistent across individuals, we identified a lesion projection zone (LPZ)/central vision ROI in each participant's data as the 100 vertices on each hemisphere with the lowest eccentricity values (200 total vertices per participant).

### FC analysis

The average time course of each ROI (LPZ, cPRL, and cURL) was extracted from the preprocessed resting-state fMRI data using in-house MATLAB scripts. FC was then calculated as Pearson's correlation between the time courses of each ROI (seed) to each voxel in the brain for each individual. The resulting whole-brain seed-based correlation maps are referred to here as “FC maps.”

### Statistical analysis

**Figure 2.**
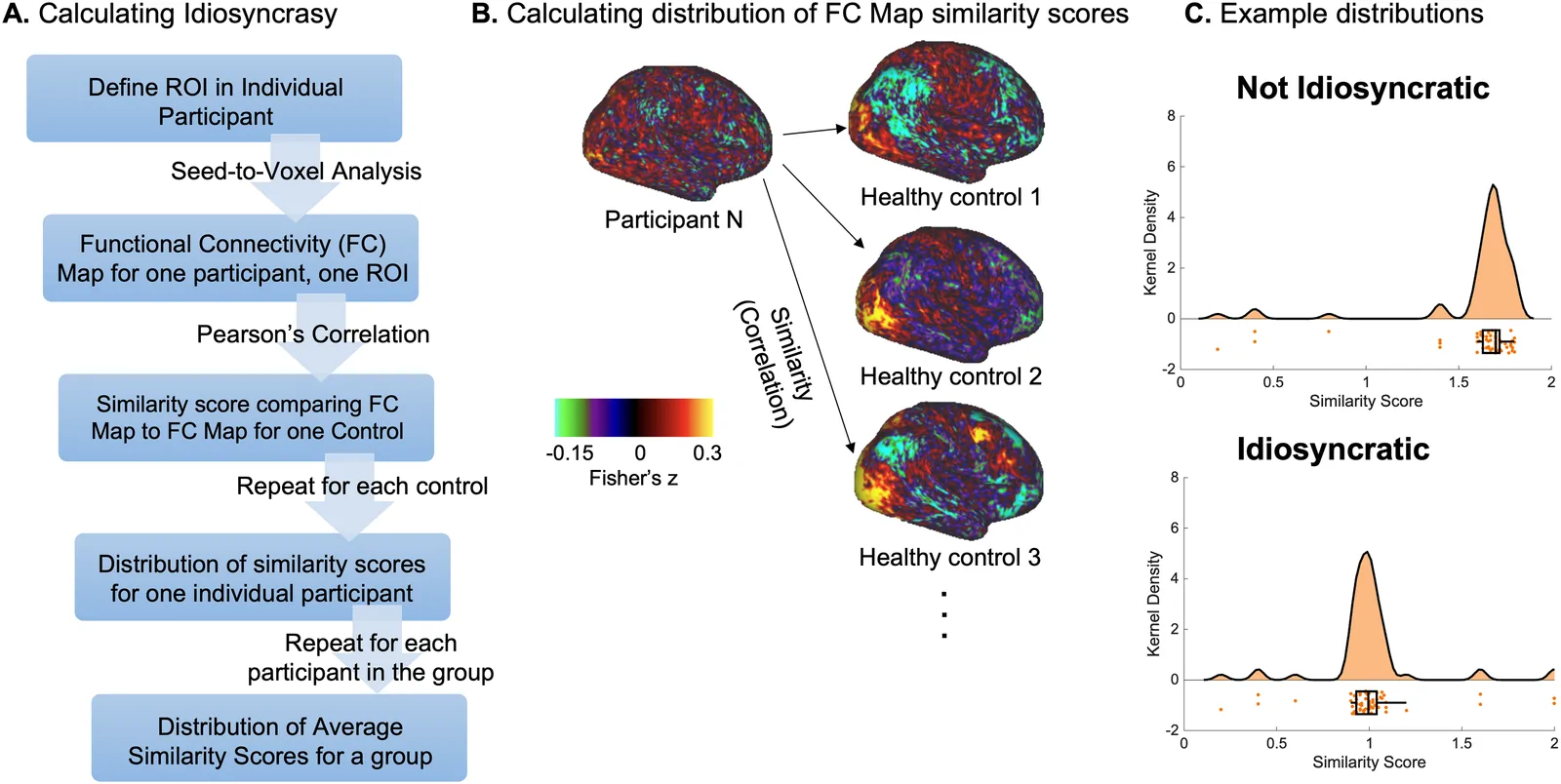
***A***, Schematic representation of how a distribution of similarity scores is calculated. ***B***, Graphical description of the “Distribution of similarity scores for one Individual participant” step in ***A***. For each ROI, for each participant, we calculate the similarity between that participant's FC map and the corresponding FC map for every HC. ***C***, Example similarity histograms for a group with more stereotypical, less-idiosyncratic patterns of connections (top), and more idiosyncratic patterns of connections (bottom).

**Figure 3.**
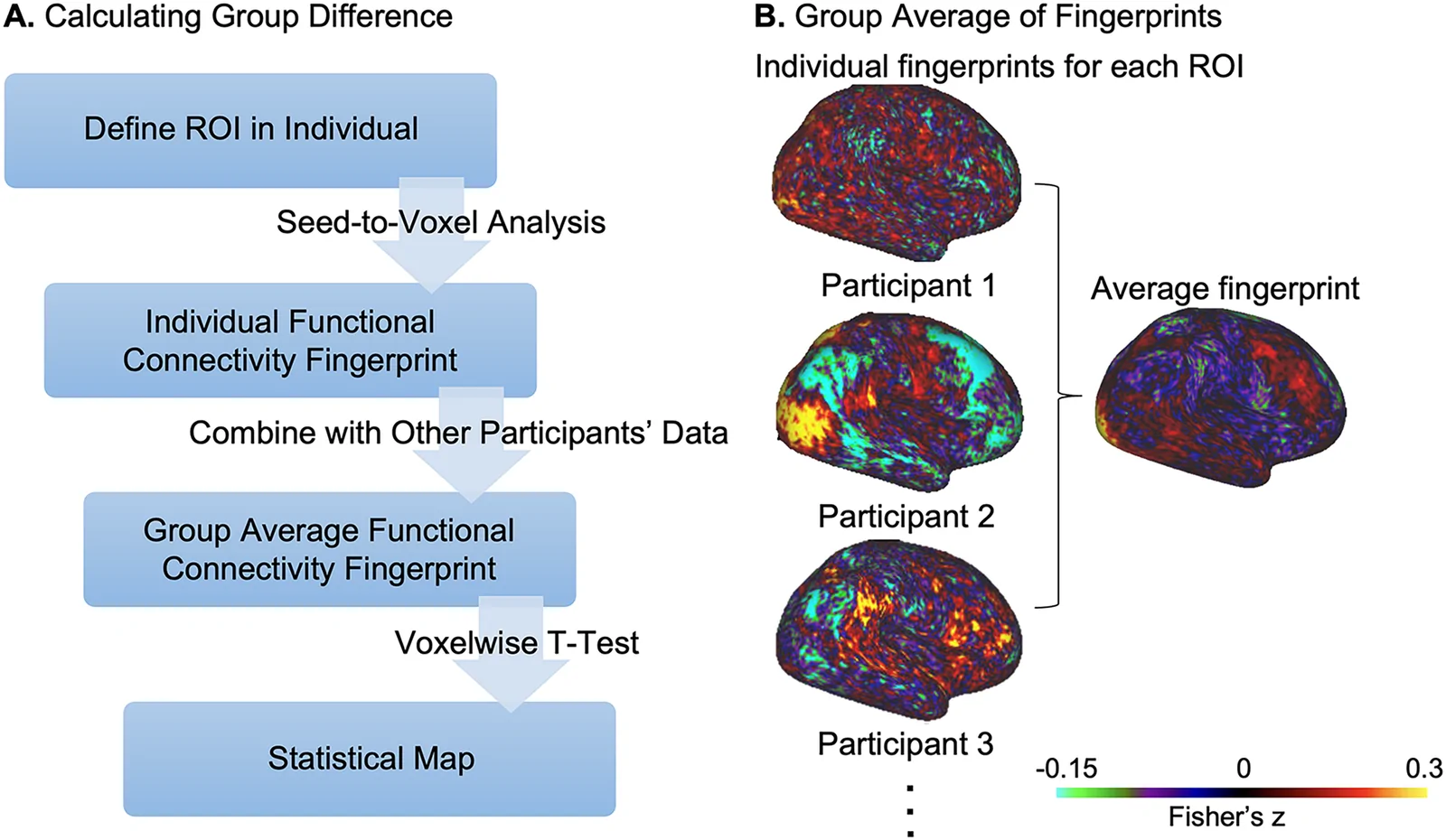
Schematic representation of (***A***) group comparison analysis and (***B***) group averages seed-to-voxel FC map analysis. FC group averages were calculated for both MD patients and healthy vision participants for LPZ, cPRL, and cURL ROIs. LPZ-seeded FC maps were defined using an ROI including the100 vertices with the lowest assigned eccentricity values around the fovea in both hemispheres in V1. PRL- and URL-seeded FC maps were calculated based on ROIs based on each MD participant's retinas.

We followed both individual-specific (Fig. 2) and group-level (Fig. 3) approaches to evaluate experience-dependent plasticity in whole-brain FC in individuals with MD.

#### Defining idiosyncrasy of a FC map

We defined retinal locations of the scotoma, PRL, and URL in individuals' MAIA images, and then we mapped them on each individual's cortex. By using these cortical ROIs, labeled LPZ, cPRL, and cURL as seeds, we performed seed-to-voxel analysis and created individual FC maps for each individual for a given seed.

We compared FC maps between two individuals by performing Pearson's correlation across all vertices (resulting in one Pearson's *r* value per participant pair). Then, we converted Pearson's correlation scores to Fisher's *z* scores and refer to this as the “similarity” of a pair of individuals' connectivity patterns.

Figure 2 illustrates the method for comparing these similarity scores. To examine distributions of similarity scores for, e.g., central vision representations, we calculated the similarity of a given participant's FC pattern for the central vision ROI to every other control participant's FC pattern to the same ROI. The mean of these similarities is then calculated for this given participant. When this value is high, it means the pattern is very similar to the typical control participant. These mean similarity scores are calculated for each participant, and the distribution of these scores across participants is plotted as a histogram.

We performed analyses of variance (ANOVAs) to test the impacts of diagnosis and ROI on similarity scores. The specific data included in models to test each hypothesis are described further in the results section, but these ANOVAs were run on the outputs of linear mixed-effect models using versions of the following formula using MATLAB's fitlme function, using main effects of ROI, diagnosis of the participant, and interaction between diagnosis of participant and ROI as fixed-effect predictors, as well as random effects for participant:

Similarity∼ROI+DiagnosisofParticipant+(DiagnosisofParticipant*ROI)+(1|Participant).

#### Group-level comparisons

We also completed a more standard group-level analysis (Fig. 3) to compare FC maps in individuals with MD to controls. For each MD participant, the PRL and URL were defined based on their own retinal data, as described above. For each healthy vision control participant, the cPRL from every MD participant was defined on that control participant's cortex, and an FC map was made for each of those ROIs. Thus, each control participant contributed multiple FC maps to the analysis, one for each cPRL. The same is true for the cURL areas. As above, the LPZ/central vision ROI was defined based in the 100 vertices in V1 corresponding to the central-most portion of vision. We used an independent-sample *t* test to compare the FC maps for ROIs between MD patients and healthy vision participants. We used a cluster-based thresholding method for group-level comparisons (Anderson and Robinson, 2001; Winkler et al., 2014). We performed cluster-based thresholding-corrected multiple comparisons for our results by using the FSL (Jenkinson et al., 2012) randomize function. We set a very liberal threshold of 1.5 for *t* contrasts so that potential effects could be observed in this group-level comparison, minimizing the possibility of a false negative statistical result.

## Results

### Central vision FC maps are more idiosyncratic than peripheral vision FC maps in healthy vision participants

To examine the individual variability of FC patterns associated with different parts of the visual field, we compared the spatial similarity of FC maps derived from central and peripheral visual representations for participants with healthy vision. We calculated the idiosyncrasy based on similarity of an individual's FC map to a group of healthy control (HC) participants, as described in Figure 2.

**Figure 4.**
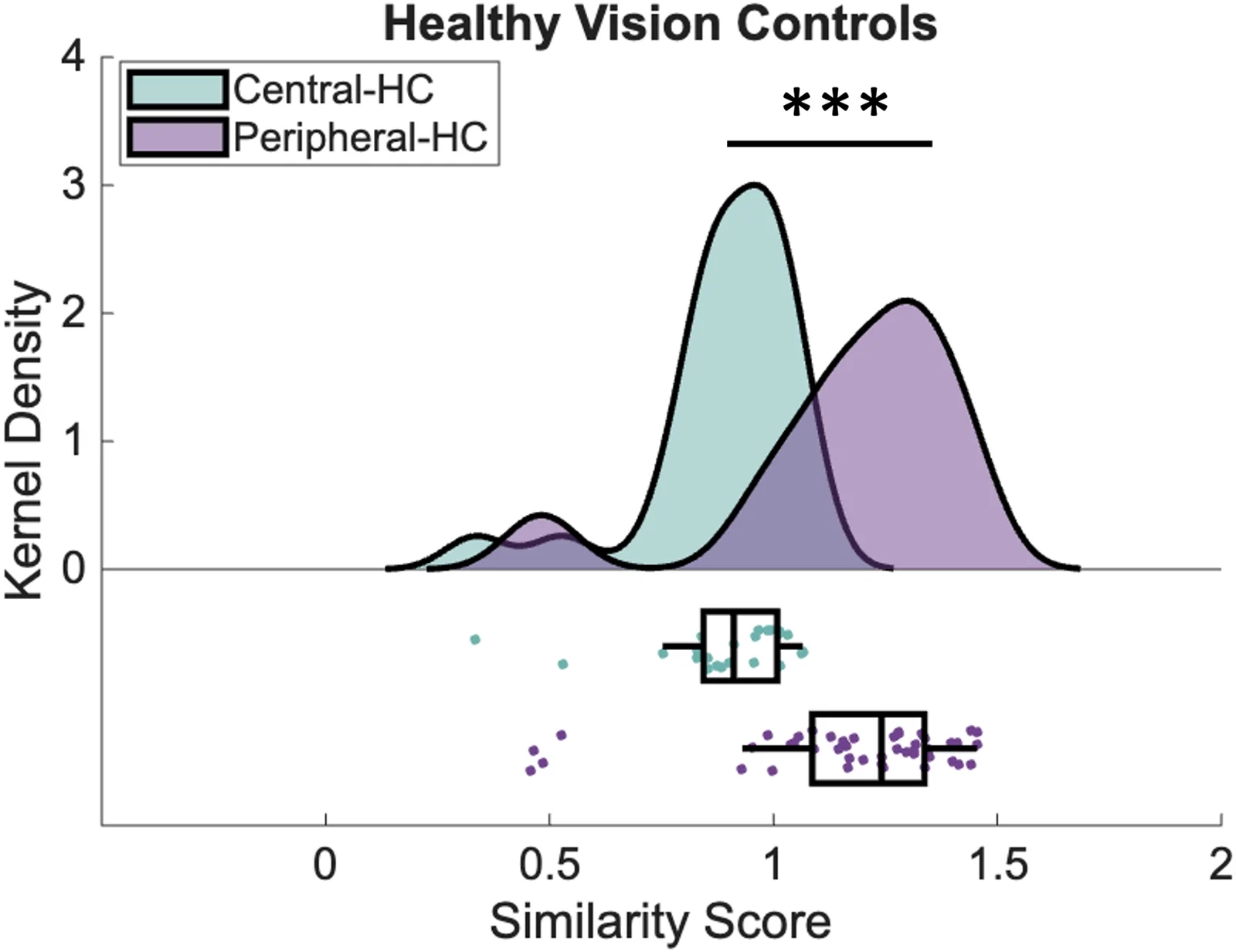
Distribution of similarity scores for FC maps seeded with cortical representations of central and peripheral vision in healthy vision individuals. Note that the maps for the central ROI (green) are significantly more idiosyncratic than for the peripheral ROI (purple) in healthy vision controls. Colored data points indicate the mean similarity score for individual participants. Histograms show the distribution across all healthy vision participants.

Our results revealed that FC maps associated with central vision exhibited significantly lower intersubject similarity compared with those derived from peripheral vision, indicating greater idiosyncrasy in the central visual field's connectivity profile (Fig. 4). ANOVA showed that the main effect of ROI (central vs peripheral) was significant for the connectivity FC maps of central and peripheral vision processing regions within healthy vision controls (*t*_(67)_ = −4.74; *p* < 0.001). Our results suggest that more attentive usage of central vision in individuals with healthy vision is associated with more idiosyncratic FC maps of central vision representations than peripheral vision representations.

### Experience with loss of sensory input is associated with no change in overall idiosyncrasy

**Figure 5.**
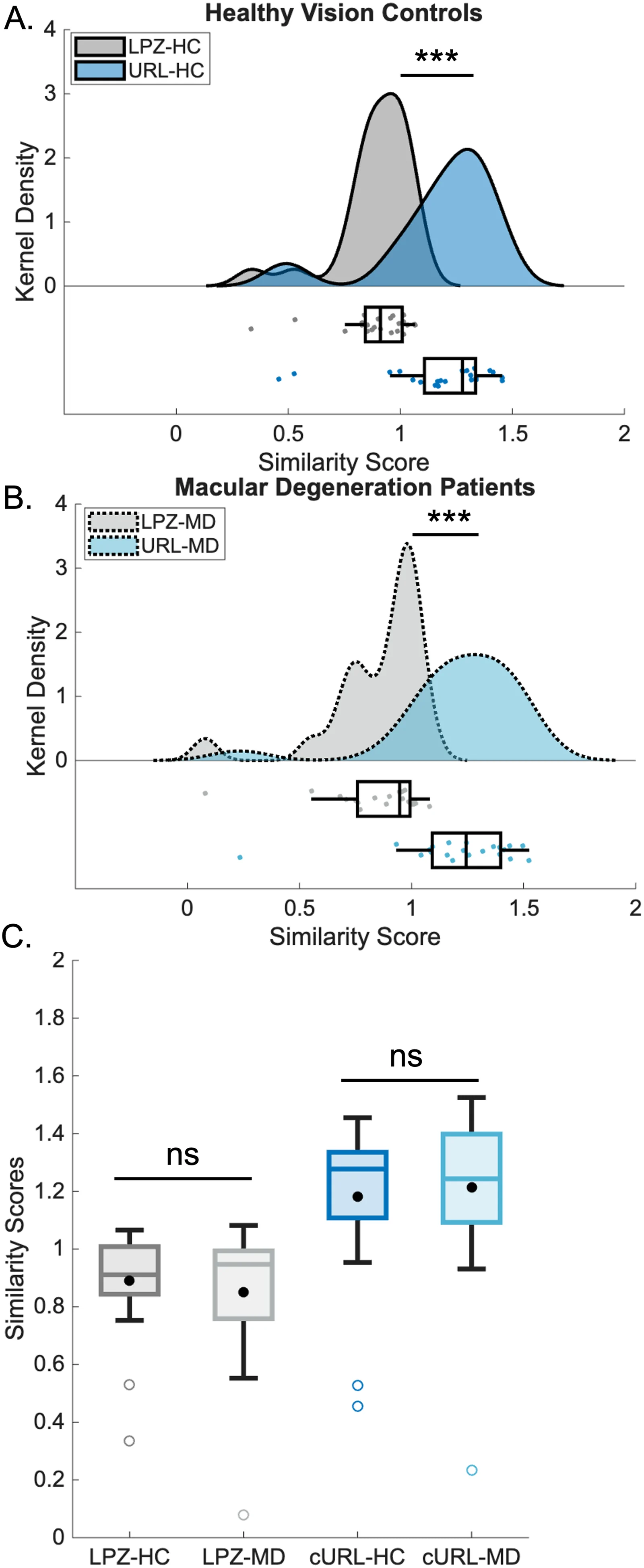
Central vision processing cortical region (LPZ)-seeded functional connections are more idiosyncratic than cURL-seeded functional connections in both MD patients and healthy vision controls. Distribution of similarity scores of LPZ- and cURL-seeded FC maps in healthy vision controls (***A***) and MD patients (***B***). ***C***, Group-level independent-sample *t* test comparisons of similarity scores did not find a difference between MD and HC groups, either for the LPZ- or the cURL-seeded FC maps. The black data point represents the group mean, and the colored horizontal line represents the group median. Error bars indicate ±1 standard error. The top and bottom bounds of each box represent the 75th and 25th percentiles, respectively. The whiskers extend to the minima and maxima data points not considered outliers. **p < 0.01.

We also examined idiosyncrasy of functional connection patterns comparing participants with central vision loss due to MD to HC. Participants with central vision loss had lost retinal input to the center of vision; LPZ is the associated portion of cortex. A control peripheral vision processing region was defined in all participants, where retinal input was similar to the PRL, but the area was not used habitually for attentive tasks; this region is called the URL, and the associated portion of primary visual cortex is called the cURL. We compared FC maps' similarity between LPZ and cURL in MD and HC participants by implementing linear mixed-effect model ANOVA (Fig. 5). We found a statistically significant main effect of ROI (*F*_(1,84)_ = 110.13; *p* < 0.001) but main effect of diagnosis (*F*_(1,84)_ = 0.31; *p* = 0.58) and interaction between ROI (LPZ, cURL) and diagnosis of the participants (MD vs HC; *F*_(1,84)_ = 2.23; *p* = 0.14) did not reach the significance level. We used Bayesian analysis to quantify the evidence in favor of the null model, where the distributions of LPZ and cURL are identical. We compared the full linear mixed-effect model including the diagnosis term with a reduced model excluding diagnosis and estimated an approximate Bayes factor using the Bayesian information criterion. The resulting Bayes factor (BF₀₁ = 29.61) provides strong evidence in favor of the null model, indicating that the data are substantially more consistent with the absence of a diagnosis effect.

We also found that LPZ- and cURL-based similarity scores differ in healthy vision individuals (Fig. 5*A*; *t*_(22)_ = −13.21; *p* < 0.001) and in MD participants (Fig. 5*B*; *t*_(20)_ = −8.03; *p* < 0.001). When we directly compare similarity scores from central vision processing region (LPZ) and peripheral vision processing region (cURL) in V1 between the individuals with central vision loss and individuals with healthy vision by using an independent-sample *t* test (Fig. 5*C*), similarity scores based on both LPZ (*t*_(42)_ = 0.65; *p* = 0.52) and cURL (*t*_(42)_ = −0.41; *p* = 0.69) are not different between groups (Fig. 5*C*). Mean (*μ*) and standard deviations (*σ*) of similarity scores of MD patients versus healthy vision controls based on ROIs were as follows: MDμ_LPZ_, 0.8511; MDσ_LPZ_, 0.2242; HCμ_LPZ_, 0.8897; HCσ_LPZ_, 0.1695; MDμ_cURL_, 1.2139; MDσ_cURL_, 0.278; HCμ_cURL_, 1.1812; HCσ_cURL_, 0.2573.

### Experience with more attentive use is associated with more idiosyncratic connection patterns in MD patients

**Figure 6.**
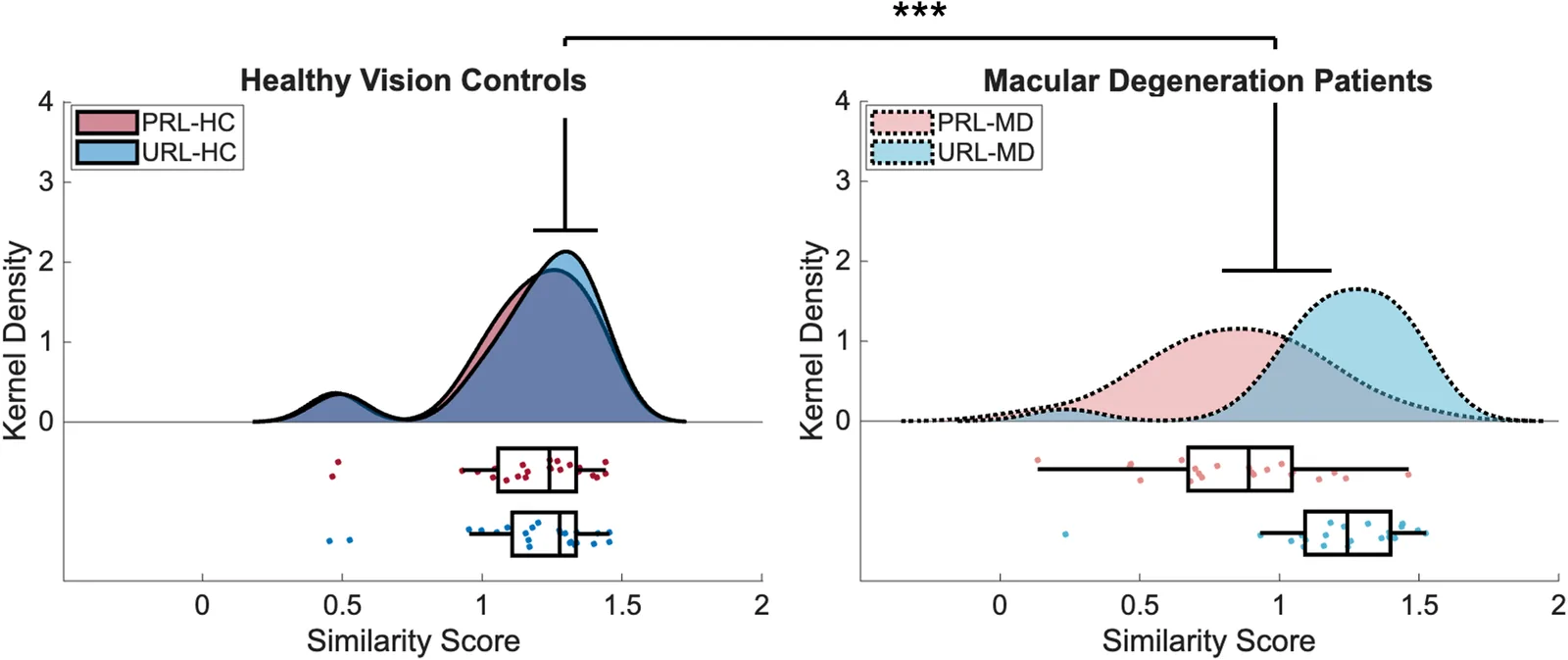
The cortical projection of the PRL has more idiosyncratic patterns of FC in MD participants. cPRL- (shown in red) and cURL-seeded (blue) FC maps' distribution of similarity scores are shown for MD patients (dashed line) and healthy vision controls (solid line). Note that more attentive use of a specific region in peripheral vision (the PRL) in individuals with central vision loss is linked to more idiosyncratic FC maps for the part of the cortex associated with that part of vision. ***p < 0.001 for the ANOVA of group by ROI.

We examined idiosyncrasy of functional connection patterns of the cortical region associated with the portion of vision that the MD participants used more, the cPRL, comparing MD to HC. As above, results from this region were compared with the control location, the cURL, where retinal input was similar to the PRL but not used habitually for attentive tasks. We analyzed individual variability in cPRL- and cURL-seeded FC patterns to evaluate sensory experience-dependent changes following more attentive usage of cPRL. Distributions of the similarity scores of MD patients and healthy vision controls are shown in Figure 6. We found that the main effect of ROI (*F*_(1,84)_ = 120.28; *p* < 0.001), main effect of diagnosis (*F*_(1,84)_ = 15.32; *p* < 0.001), and the interactions between ROI and diagnosis of the participants between cPRL- and cURL-seeded FC maps (*F*_(1,84)_ = 54.91; *p* < 0.001) are statistically significant. Mean (*μ*) and standard deviations (*σ*) of similarity scores of MD patients versus healthy vision controls based on ROIs were as follows: MDμ_cPRL_, 0.8384; MDσ_cPRL_, 0.3091; HCμ_cPRL_, 1.1566; HCσ_cPRL_, 0.2586; MDμ_cURL_, 1.2139; MDσ_cURL_, 0.278; HCμ_cURL_, 1.1812; HCσ_cURL_, 0.2573.

**Figure 7.**
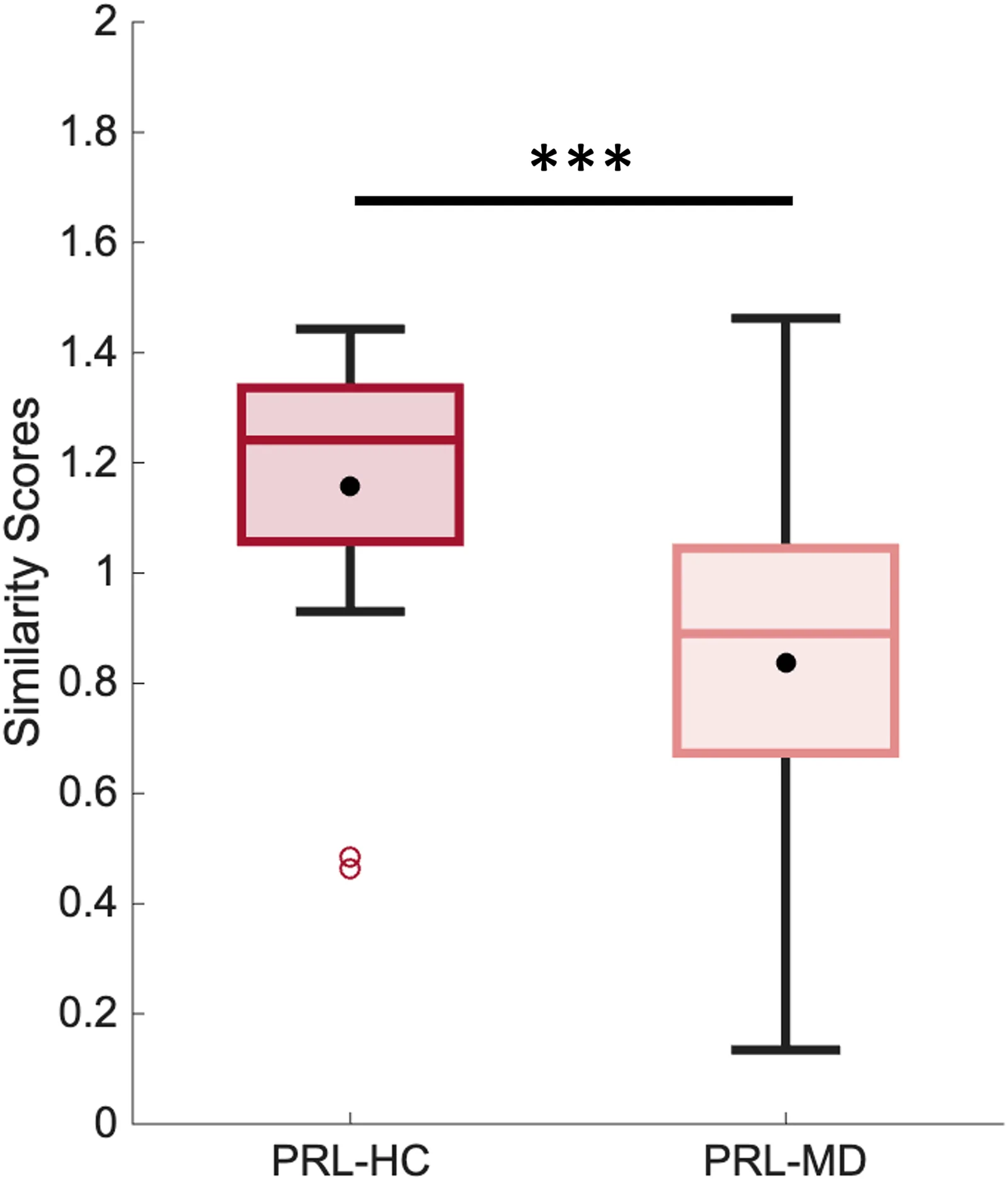
Cortical projection of the cPRL-seeded FC maps is more idiosyncratic in MD patients than in the healthy vision control group. Group-level independent-sample *t* test comparisons of similarity scores showed that cPRL-seeded (*t*_(42)_ = 3.72; *p* < 0.001) FC maps have lower similarity scores (more idiosyncratic) for MD patients than healthy vision controls. Colored data points indicate individual measurements. The black data point represents the group average, and the colored horizontal line represents the group median. Error bars indicate ±1 standard error. Top and bottom bounds of each box represent the 75th and 25th percentiles, respectively. The whiskers extend to the minima and maxima data points, not considered outliers. ***p < 0.001.

Next, we compared similarity scores from only the cPRL region, using an independent samples *t* test. Our results showed that the similarity of cPRL-seeded functional connections was higher in HC than in MD participants (Fig. 7; *t*_(42)_ = 3.72; *p* < 0.001). These results indicate that experience with increased usage is associated with more idiosyncratic functional connection patterns.

### FC patterns are more idiosyncratic the more stable and precise a patient's use of the PRL

**Figure 8.**
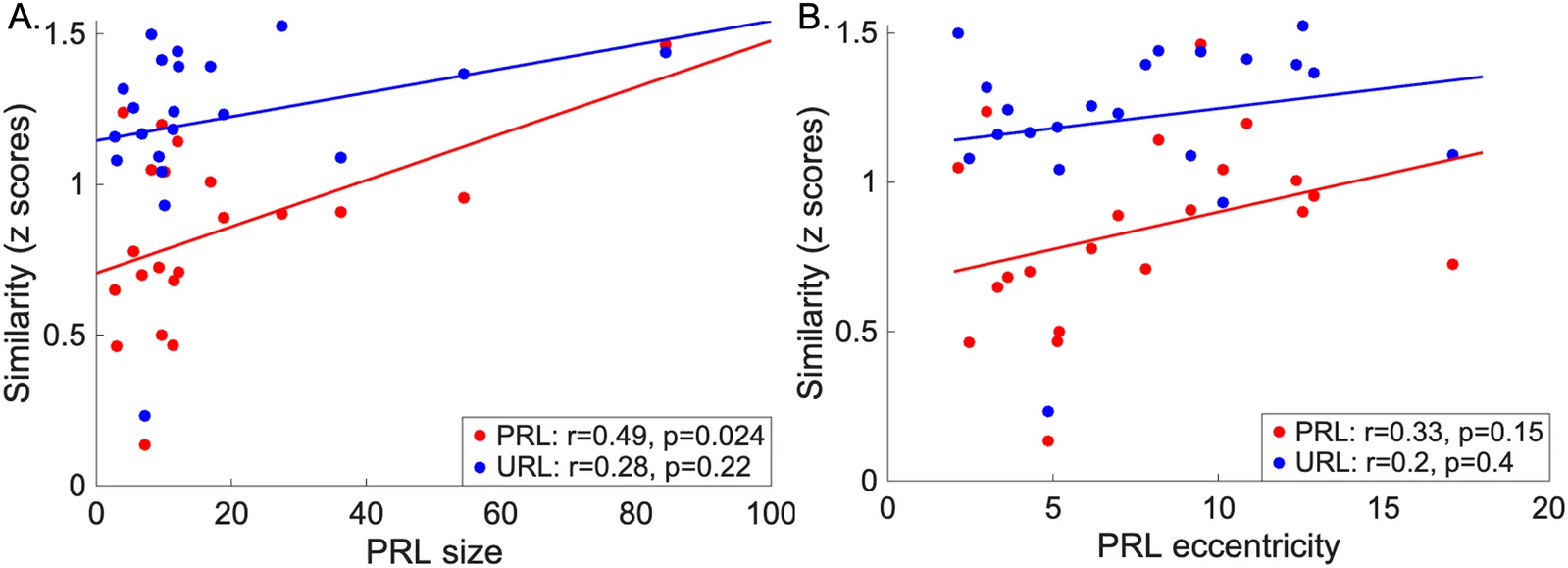
Correlations between cPRL- and cURL-based similarity scores and PRL size and eccentricity in MD patients. When the PRL is small in size, the similarity of FC maps based on the cortical projection of the PRL is less similar to HC (red, *r* = 0.49; *p* = 0.024). The URL size is not significantly correlated with cURL-based similarity scores in MD patients (blue). PRL or URL eccentricity to the center is not correlated with similarity scores in MD patients.

We also assessed how the idiosyncrasy of functional connections depended on known differences in individuals' experiences (Fig. 8). Because of participants' different personal experiences and daily needs, as well as their variable scotoma shape and severity, their attentive usage of a PRL is likely to differ from person to person. People who must use a nonfoveal PRL have improved performance on daily tasks when they are able to maintain stable fixation at that PRL (Crossland et al., 2011). The stability of this fixation is measured using the “PRL size” measured as the “bivariate contour ellipse area” (Crossland et al., 2011)—a small PRL size means fixation points fall more often within a small distance from the target. Thus, participants who have a smaller PRL size, all else being equal, are better at maintaining attention to their PRL.

We found that the PRL size is positively correlated with the similarity of the cPRL (*r* = 0.49; *p* = 0.024). It was not correlated with the similarity of the cURL (Fig. 8*A*; *r* = 0.28; *p* = 0.22). This finding is consistent with the idea that the cortical representation of a more attentively used retinal area has more idiosyncratic connections than a less attentively used area.

PRLs that are more eccentric also tend to have larger PRL sizes, consistent with generally poorer fixation stability for more peripheral locations (Whittaker et al., 1988). Thus, we also assessed the relationship between PRL eccentricity and similarity scores. PRL eccentricity did not significantly correlate with similarity of the cPRL (Fig. 8*B*; *r* = 0.33; *p* = 0.15), suggesting that the relationship between PRL size and similarity scores is not strongly driven by eccentricity. However, treating eccentricity as a covariate in the relationship between PRL size to cPRL similarity leads to a partial correlation of *r* = 0.4133 and *p* = 0.0701, reducing the size of the effect. Our results are generally consistent with the idea that more attentive usage (smaller PRL size due to a better fixation) is associated with less similarity in the cPRL-based connections, but not in cURL-based connections, though the strength of evidence is relatively weak since the effect is no longer significant after covarying eccentricity.

### In participants with central vision loss, FC patterns of PRL are more like FC patterns of LPZ

**Figure 9.**
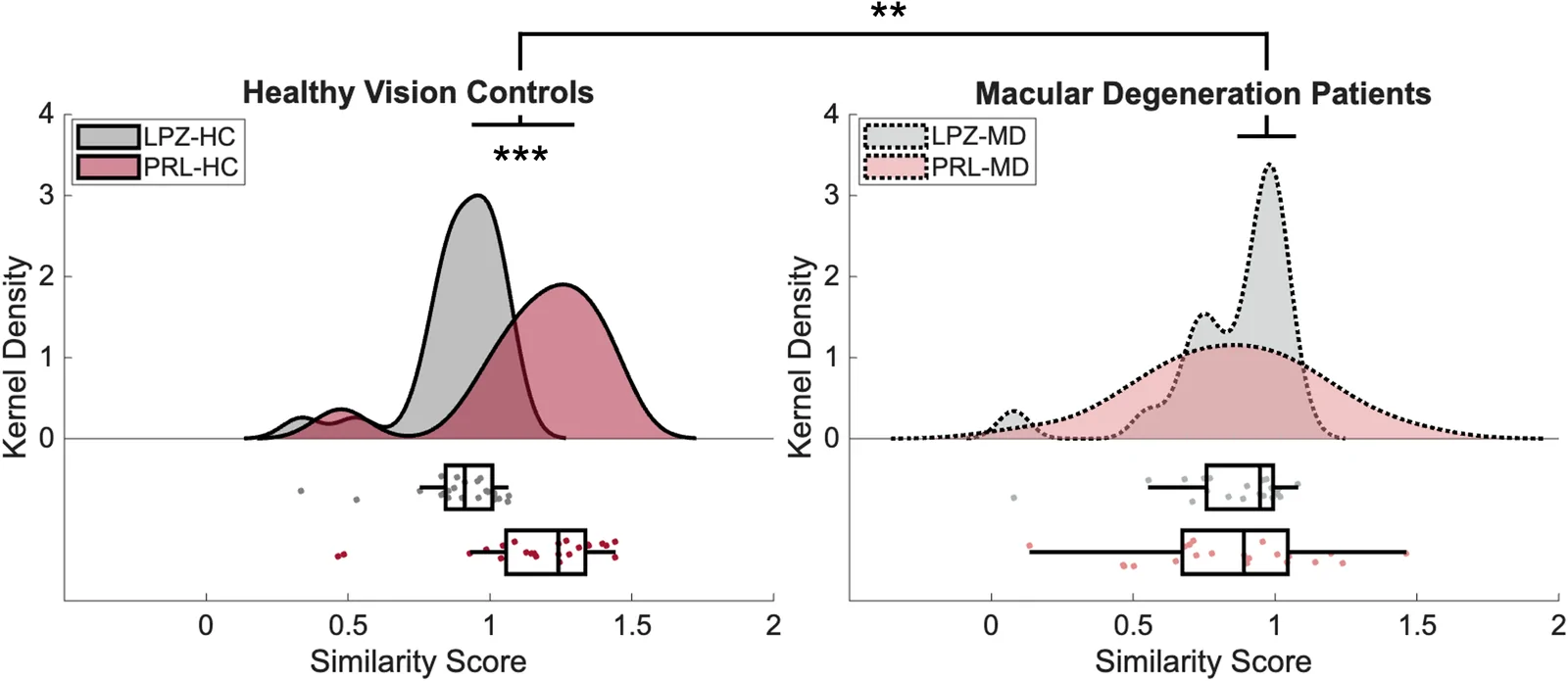
In MD patients, the cPRL has a pattern of idiosyncrasy like that in central vision. LPZ- and cPRL-seeded FC maps' similarity score distributions are significantly different between healthy vision controls and MD patients. Interaction between ROI and participant diagnosis is statistically significant (*F*_(1,84)_ = 7.52; *p* = 0.0075). LPZ- (shown in gray) and cPRL-seeded (shown in red) FC maps' distribution of similarity scores are shown for MD patients (dashed line) and healthy vision controls (solid line). ***p < 0.001 for the ANOVA of group by ROI.

In order to identify whether the idiosyncrasies of FC maps for the PRL were becoming more like those of central vision after MD, we compared the distributions of similarities for LPZ and cPRL-seeded FC patterns in MD participants and controls (Fig. 9). These are the same values as shown in Figures 3 and 4, just directly compared here. In HC, LPZ showed significantly more idiosyncratic connections than PRL (*t*_(22)_ = −11.60; *p* < 0.001), but this pattern did not hold in MD patients (*t*_(20)_ = 0.18; *p* = 0.86). Interaction between ROI and participant diagnosis was statistically significant (*F*_(1,84)_ = 7.52; *p* = 0.0075). Our results indicate that increased use of peripheral vision in people with central vision loss makes their patterns of functional connections more idiosyncratic and more comparable to the idiosyncratic functional connections that are typical for central vision. These findings are consistent with the idea that as we use specific functional connections more, they become more idiosyncratic.

### Standard group comparisons showed limited evidence for plasticity after central vision loss

**Figure 10.**
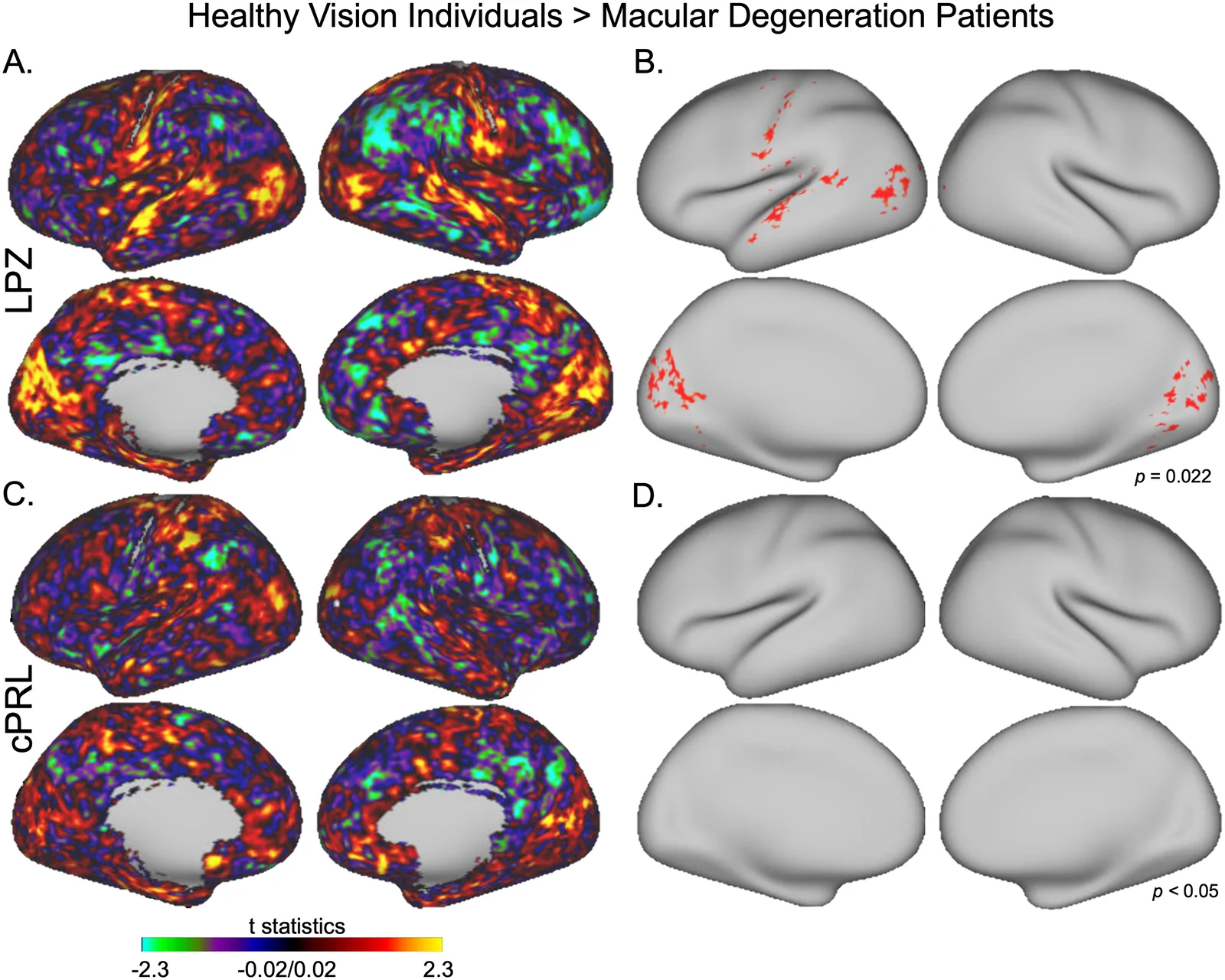
Group-wise comparison of whole-brain FC maps for MD patients versus healthy vision control groups. Group-level comparisons showed limited evidence for plasticity, when measured as a mean difference between groups. ***A, C***, Independent-sample *t* test scores between healthy vision individuals and MD patients for whole-brain FC maps for LPZ (***A***) and cPRL (***C***). Brighter colors represent more significant results. ***B, D***, FWE-corrected *p* values between healthy vision individuals and MD patients for LPZ (***B***) and cPRL (***D***). ***B***, The healthy vision control group showed stronger FC in HC than MD participants to a cluster including temporo–parieto–occipital regions (cluster size, 6,031; FWE-corrected *p* = 0.022). Group-level analysis results were projected onto to 32k cifti space template. Note that there are no significant voxels in ***D***, indicating no significant between-group differences in connection to the cortical projection of the PRL, when measured this way.

Standard group-level comparisons can reveal differences between groups; however, individual variability is high in the MD patient population. We analyzed standard group-level differences between the MD and HC groups to test outcomes when we ignore individual variability. Independent-sample *t* statistics for LPZ and cURL regions are shown in Figure 10. Our group-level LPZ, cPRL, and cURL-seeded FC map comparisons revealed that only the LPZ FC map is different between the groups; healthy vision controls have stronger FC to a cluster in the temporo–parieto–occipital cortex compared with MD patients (Fig. 10*B*; cluster size, 6,031; FWE-corrected *p* = 0.022). Figure 10*B* shows that MD patients who have central vision loss have weaker functional connections from the central vision processing region to regions within temporal, parietal, and occipital lobes. This is consistent with previous findings in those who lost vision in adulthood (Sabbah et al., 2017; Fleming et al., 2024) as well as in those who were born without vision (Striem-Amit et al., 2015) and suggests that loss of use of a brain area due to sensory deprivation results in some connectivity decreases that are consistent across people. However, using this standard method, we could not detect any group difference in functional connections of more attentive usage of a specific area in periphery (PRL) between MD patients and healthy vision controls (Fig. 10*C*,*D*). Thus, the standard group comparison method showed limited evidence for plasticity of functional connections to the deprived brain area and no evidence for plasticity of functional connections to the “more attended” brain area.

## Discussion

In participants with healthy vision—who preferentially attend to central vision—central vision representations had more idiosyncratic cortical connection patterns than peripheral vision. In participants with central vision loss—who preferentially attend to their PRL—the representations of the PRL had more idiosyncratic cortical connection patterns than other parts of peripheral vision. These patterns are more idiosyncratic the more stable their use of the PRL. Together, these results suggest that cortical regions that experience frequent attentive use develop idiosyncratic cortical connections.

### Central vision FC maps are more idiosyncratic than peripheral vision FC maps

Central and peripheral visions have different functional roles. High acuity visual information from central vision is critical for everyday activities like reading and other daily tasks requiring focused attention (Pelli et al., 2007; Larson and Loschky, 2009; Yoo and Chong, 2012), whereas peripheral vision is essential for tasks like visual search and getting the gist of the scene (Larson and Loschky, 2009). These behavioral differences are mirrored by brain connections: higher-order brain areas are more strongly connected to V1 representations in the central than peripheral visual fields (Griffis et al., 2015; Zhaoping, 2017; Sims et al., 2021).

Attention networks have more idiosyncratic patterns of connections than visual cortical networks (Mueller et al., 2013). Consistent with this pattern, in healthy participants, connections to central representations in V1 (associated with daily attention-demanding tasks) are more idiosyncratic than connections to peripheral representations. Furthermore, some degree of this region-to-region difference in connection patterns is due to experience, not solely evolutionary pressures, since the PRL showed more idiosyncratic connections only in patients with vision loss.

### Idiosyncratic connection patterns reflect use-dependent plasticity in adulthood

Our findings provide new evidence that functional connections of V1 retain the capacity for experience-dependent plasticity well into adulthood. This capacity has been the subject of controversy. For example, several studies have shown that there is very limited, if any, shift in V1 receptive field locations with experience (Smirnakis et al., 2005; Masuda et al., 2008; Wandell and Smirnakis, 2009; Baseler et al., 2011; but see Gilbert and Li, 2012, but see Gilbert and Li, 2012; Abe et al., 2015; Haak et al., 2015). Studies that examine functional connections to V1 after loss of vision have shown preserved patterns of local connections in patients with vision loss (Butt et al., 2013; Haak et al., 2016).

Previous studies using standard analytical approaches have shown that patients with central vision loss have modestly stronger long-range connections between peripheral early visual cortex (PRL) and higher-order regions involved in attention and oculomotor control (Little et al., 2008; Sabbah et al., 2017), though this effect was not significant here (Fig. 10*D*). A previous study from our team (Fleming et al., 2024) found considerable subject-to-subject variability in the strength of connections between the PRL and some cortical regions (e.g., FEF) in the patient group, but no statistical difference from the control group, a result which inspired the current study. Our data are thus consistent with studies showing limited plasticity in the visual cortex in adulthood using standard approaches. However, by taking individual differences into account, using our idiosyncrasy approach, we quantify plastic changes that are different person to person, providing clear evidence that functional connections of V1 are plastic in adulthood, and that increased attentive usage likely drives that plasticity. One potential contributor to the controversy regarding plasticity is that most studies in humans have compared means of one group to another, rather than taking individual differences into account. This individualized approach allows more nuanced identification of plasticity and may in the future allow tailoring interventions and rehabilitation strategies to each individual's unique profile.

### Sensory deprivation is associated with no change in idiosyncrasy

Whereas our data show strong evidence that connection patterns become more idiosyncratic with attentive use, we do not see evidence that they are less idiosyncratic following lack of use (Fig. 5*C*). This is surprising, given that the biggest experience change patients shared was a loss of central vision.

Patients did show modest declines in connection strength between LPZ to several brain areas (relative to the mean of healthy vision controls; Fig. 10*B*). Conversely, strengths of connections to the cPRL did not significantly differ. This pattern of results suggests that while increased attentive use may lead to more idiosyncratic patterns of connections, decreased use leads instead to weakening connections, without changing the idiosyncrasy of the whole-brain patterns.

These results suggest that the plasticity mechanisms differ for increased attentive use versus decreased attentive use (or sensory loss). Increased attentive use may strengthen some connections and weaken others, as needed for the specific and idiosyncratic functions needed for the attended task. On the other hand, visual input loss and decreased attentive use may lead to overall weakening of connections and no appreciable change in idiosyncrasy of the patterns of connections.

Despite the evidence of limited plasticity in receptive fields in the LPZ (Smirnakis et al., 2005; Wandell and Smirnakis, 2009; Baseler et al., 2011; Haak et al., 2015), the LPZ shows BOLD activity during performance of attention-demanding tasks (Baker et al., 2005; Masuda et al., 2008; Dilks et al., 2009). This activity, however, is absent during passive fixation (Masuda et al., 2008, 2021), is not limited to a specific portion of the retina (Dilks et al., 2009), and occurs during nonvisual tasks (Masuda et al., 2021). This suggests that LPZ activity in patients with central vision loss results from something other than direct input from the thalamus, perhaps from plasticity of lateral or top–down connections (Gilbert and Li, 2013).

### Plausible mechanisms underlying increased idiosyncrasy of PRL-based FC patterns

The strength of neuronal connections often follow Hebb's rule, “cells that fire together wire together” (Hebb, 1949; Buonomano and Merzenich, 1998). Due to the retinotopic nature of V1, the repeated use of a specific area of the retina (e.g., the PRL) leads to repeated activation of its cortical representation as well as downstream targets. When an attention-demanding task is performed, there is likely to be near-simultaneous activity of the cortical representations of the visual stimulus and attentional control networks like the frontoparietal and dorsal attention systems (Kastner and Ungerleider, 2000; Corbetta and Shulman, 2002). Thus, repeated performance of attention-demanding tasks using a PRL is likely to result, through Hebbian plasticity, in selective strengthening of the connections between the cortical representation of the PRL and downstream areas. Some studies have observed an increase in cortical thickness in the cortical representation of the PRL (Burge et al., 2016; Plank et al., 2021; Defenderfer et al., 2023).

The specific tasks and strategies for compensating for vision loss likely differ from participant to participant and thus would impact different brain areas in different people. People who were born blind show highly variable FC patterns to V1, and the connection to frontal areas is stronger with increased education level, consistent with this idea (Sen et al., 2022). Because attention networks themselves exhibit high interindividual variability (Mueller et al., 2013), strengthening the patterns of connections between the cPRL to individual-specific attention networks would further amplify the idiosyncrasy of whole-brain functional connections.

### Ruling out potential confounding factors

Participants closing their eyes versus having their eyes open or fixating versus moving their eyes influences intrinsic connectivity in resting-state data (Patriat et al., 2013). To equate the retinal contrast of participants, scans were collected in darkness. Because fixation is inherently unstable in patients, we permitted free eye movements in both the MD and control groups to reduce systematic differences between them. Critically, the changes in connection patterns we observed were found in the cortical projection of the PRL (not the URL) meaning that the observed differences stem from factors that are specific to that location. This approach offers a within-subject control.

Each patient develops a PRL at a different retinal location, due to many factors including the extent and shape of their retinal scotoma. Perceptual performance varies across visual field locations in people with intact vision (Carrasco et al., 2001, 2004). Thus, differences in the function of different people's PRLs could arise from a range of reasons. One of the benefits of our analytical approach is that it takes these different PRL locations into consideration and allows us to examine connectivity patterns to a specific swath of the cortex corresponding to an individual patient's PRL.

Other factors may also contribute to the subject-to-subject variability of FC patterns. Performance of a task just prior (Waites et al., 2005), caffeine intake (Barry et al., 2005; Wong et al., 2012), and falling asleep (Deco et al., 2014) can bias resting-state measurements. These factors should not differentially impact the PRL versus the URL, however.

We examined V1 largely because its retinotopic nature allows identification of the specific swaths of the cortex that will have undergone shifts in activity following development of a PRL. This finding may generalize to other parts of the cortex, but future work in different populations would be required in order to examine this carefully. For example, effects in the somatosensory or motor cortex might be identified by examining amputees or musicians.
